# Influence of Anaesthesia on Harvesting the Semitendinosus Tendon for Anterior Cruciate Ligament Replacement

**DOI:** 10.7759/cureus.30791

**Published:** 2022-10-28

**Authors:** Remy Rees-Goddard, Kim Borsky, Tobias Tessmann, Thomas Wolf, Thomas Boeker-Blum, Michael Borsky

**Affiliations:** 1 Orthopaedic Surgery, Remy Rees-Goddard, Isle of Wight, GBR; 2 Plastic Surgery, Oxford School of Surgery, Oxford, GBR; 3 Anaesthesiology, Spital Lachen, Lachen, CHE; 4 Knee Surgery Division, Etzelclinic, Pfaeffikon, CHE

**Keywords:** surgical anaesthesia, ortho surgery, tendon graft, knee injuries, anterior cruciate ligament (acl) reconstruction

## Abstract

Aim

The aim of the study was to investigate if the mode of anesthesia is a relevant factor influencing the length of the semitendinosus tendon harvested for anterior cruciate ligament (ACL) replacement.

Methods

Patients undergoing ACL replacement were given the choice between spinal and general anesthesia. If general anesthesia was chosen, a short relaxation period was applied while harvesting the Semitendinosus (ST) tendon. The data for tendon length, the folding factor, and the diameter of the graft were collected. Except for redo procedures and ACL replacement with allograft all patients undergoing an ACL replacement were enrolled.

Results

Thirty-one patients with general anesthesia (GA) and 14 patients with spinal anesthesia (SA) were examined. The ST tendon length was 29.03 cm ± 2.6 cm in the GA group and 25.4 cm ± 2.70 in the SA group (t value 4.245, p=0.0001). The ST tendon could be quadrupled in 23 patients in the GA and 6 patients in the SA group (χ2=4.13, p=0.04). The graft diameter obtained was 8.53 mm ± 0.62 mm in the GA group and 7.71 mm ± 0.47 mm in the SA group (t value 4.885, p<0.0001).

Conclusion

General anesthesia with short-time relaxation while harvesting the ST tendon for ACL grafting allows to harvest longer ST tendons and consequently yields better results regarding the graft dimensions and should be offered to patients as a first-choice procedure. There is no other paper published yet, analyzing this relationship and if validated on a larger cohort, this might change clinical practice.

## Introduction

Failure rates of anterior cruciate ligament (ACL) repairs have been extensively discussed in the literature and one of the aspects repeatedly analyzed is the graft diameter, showing that the tendon diameter is an important factor influencing the failure rate and that a bigger diameter is associated with lower failure rates [1-10]. Our hypothesis was that harvesting the tendon under general anesthesia, with short time relaxation while harvesting the tendon, will result in a longer tendon graft, leading to a bigger diameter compared to spinal anesthesia. Obviously, the bigger diameter should result from the fact, that a longer tendon graft can more frequently be used quadrupled than a shorter one which is used tripled or even doubled. The purpose of this study was to investigate the influence of the mode of anesthesia in relation to the length of the semitendinosus tendon harvested for ACL replacement. There is currently no gold standard regarding the choice of anesthesia for these procedures and the decision is based on patient-specific or healthcare system-related factors (cost, availability, etc.). To the best of our knowledge, no literature is available at present researching the influence of the mode of anesthesia on the quality of a tendon autograft.

## Materials and methods

Patients

In a prospective comparative cohort study from August 2018 until January 2019 45 patients undergoing primary ACL replacement by semitendinosus (ST) tendon graft were enrolled. Between July 2018 and December 2019 45 patients were operated on fulfilling the inclusion criteria. Ten redo procedures were excluded as well as 15 allograft procedures. Informed consent was gained from all patients before undergoing a procedure. All procedures performed involving human participants were in accordance with the ethical standards of the institutional board. This study met the criteria for service evaluation, which according to Swiss law does not require specific ethical approval [11].

Surgical technique

The choice of anesthesia was made by the patient after consultation with the anesthesiologist prior to the operation. All procedures were done by the same surgeon. The ST tendon was harvested via a short popliteal incision [12]. Patients were placed supine with the leg flexed at the hip and knee to allow access to the popliteal fossa. Through a small transverse incision, the semitendinosus tendon is identified. An open tendon stripper is looped around the tendon to detach it from its proximal origin with the leg in extension. Through a closed stripper, the distal insertion is then detached. The same standard-size strippers were used for all patients. In all patients, a tourniquet was only applied after harvesting the tendon. Femoral fixation was obtained by an ACL tight rope, the tibial fixation by an interference screw. The length required for a quadrupled graft was at least 27 cm. Also, the desired graft diameter was 8 mm or more. The size of the graft diameter was measured with the Arthrex sizing block in 0.5cm increments. The smallest diameter through which the folded tendon could pass was considered the graft diameter. Where a quadrupled graft couldn’t be obtained the tendon was tripled in order to get a diameter as large as possible. The tripled tendon was prepared in a four-rip-stop suture technique as described by Yoo [13].

Anaesthetic technique

Patients received oral pre-medication with either midazolam 7,5 mg, melatonin 5 mg, or oral clonidine 150 mcg depending on their individual co-morbidities 60 to 90 minutes prior to the start of the anaesthesiologic procedure. Routine monitoring of vital signs and intravenous access was established in all patients. Patients scheduled for general anesthesia received an induction dose of propofol (1 to 2.5mg per kg bodyweight), fentanyl 2 mcg per kg body weight, and rocuronium 0.6 mg per kg bodyweight. After the airway had been secured, controlled mechanical ventilation was started and general anesthesia was maintained with either a continuous propofol-remifentanil infusion or sevoflurane. 

In all patients receiving general anesthesia neuro-muscular monitoring was performed using the “ToFscan“ (produced by idmed, Marseille, France), stimulating the ulnar nerve with a train-of-four pattern consisting of four electric stimuli of 60 mA for 0.2ms each at a 2 Hz frequency. The resulting adductor pollicis muscle contraction was registered using a 3D-acceleromyography sensor fixed to the thumb. Careful positioning of the hand and forearm guaranteed the thumb‘s free range of motion. Complete neuro-muscular blockade (measured as a TOF response of 0) was ensured by the time of harvesting the tendon. Likewise, prior to the end of the surgery, the complete restitution of neuro-muscular integrity was ensured with a TOF 4/4 ratio of >90%. If necessary, antagonization using sugammadex 2 mg per kg bodyweight was administered.

Patients scheduled for spinal anesthesia were positioned in lateral decubitus position, resting on the side to be operated on. The spinal puncture was carried out as usual: after thorough skin disinfection and sterile draping, local skin anesthesia with 20 to 50 mg lidocaine 1% was applied and a 25G or 27G pencil-point needle was advanced through median or paramedian approach at the L3/4 or L4/5 paravertrebral spaces until CSF could be attained. 10 mg to 12.5 mg of 0.5% hyperbaric bupivacaine depending on the patient’s body height was administered and the lateral positioning was maintained as long as feasible with a minimum of 10 minutes. Light intravenous sedation using propofol and/or remifentanil and acoustic shielding through headphones playing music were offered to all patients. Since the ulnar nerve remained unaffected by the spinal anesthetic, "ToFscan" to assess for potential partial muscle relaxation resulting from the spinal anesthetic could not be used in the spinal cohort. Care was taken to avoid the administration of any drugs which might interfere with neuro-muscular integrity, e.g. no magnesium sulfate was administered. There were two patients in whom spinal anesthesia could not be administered due to technical or anatomical difficulties or in whom the analgetic effect was deemed to be insufficient for surgery, thus requiring additional induction of general anesthesia. These patients’ data were excluded from further analysis.

Statistical analysis

Statistical analysis was performed by student t-test for the qualitative parameters and Fischer exact test for the quantitative parameters. A significant difference was considered at the 0.05 level.

## Results

Between July 2018 and December 2019 45 patients were operated on fulfilling the inclusion criteria. There were 31 Patients in the general anesthesia (GA) and 14 patients in the spinal anesthesia group (SA). Patient and tendon characteristics are summarised in Table 1.

**Table 1:** Patient and tendon characteristics Baseline demographic data and tendon characteristics of the study population. P-values assess differences between the two patient cohorts.

	General anaesthesia	Spinal anaesthesia	p-value
n	31	14	
Age, mean ±SD	29.03 ± 12.44	25.43 ± 11.00	0.34
Height cm, mean ±SD	172.61 ± 6.21	168.50 ± 6.73	0.26
Weight kg, mean ±SD	68.21 ± 12.11	75.29 ± 13.68	0.11
Tendon length cm, mean ±SD	29.03 ± 2.6	25.4 ± 2.70	0.0001
Tendon diameter mm, mean ±SD	8.53 ± 0.62	7.71 ± 0.47	<0.0001
cm=centimetre, kg=kilogram, mm=millimeter, SD=standard deviation			

**Figure 1:**
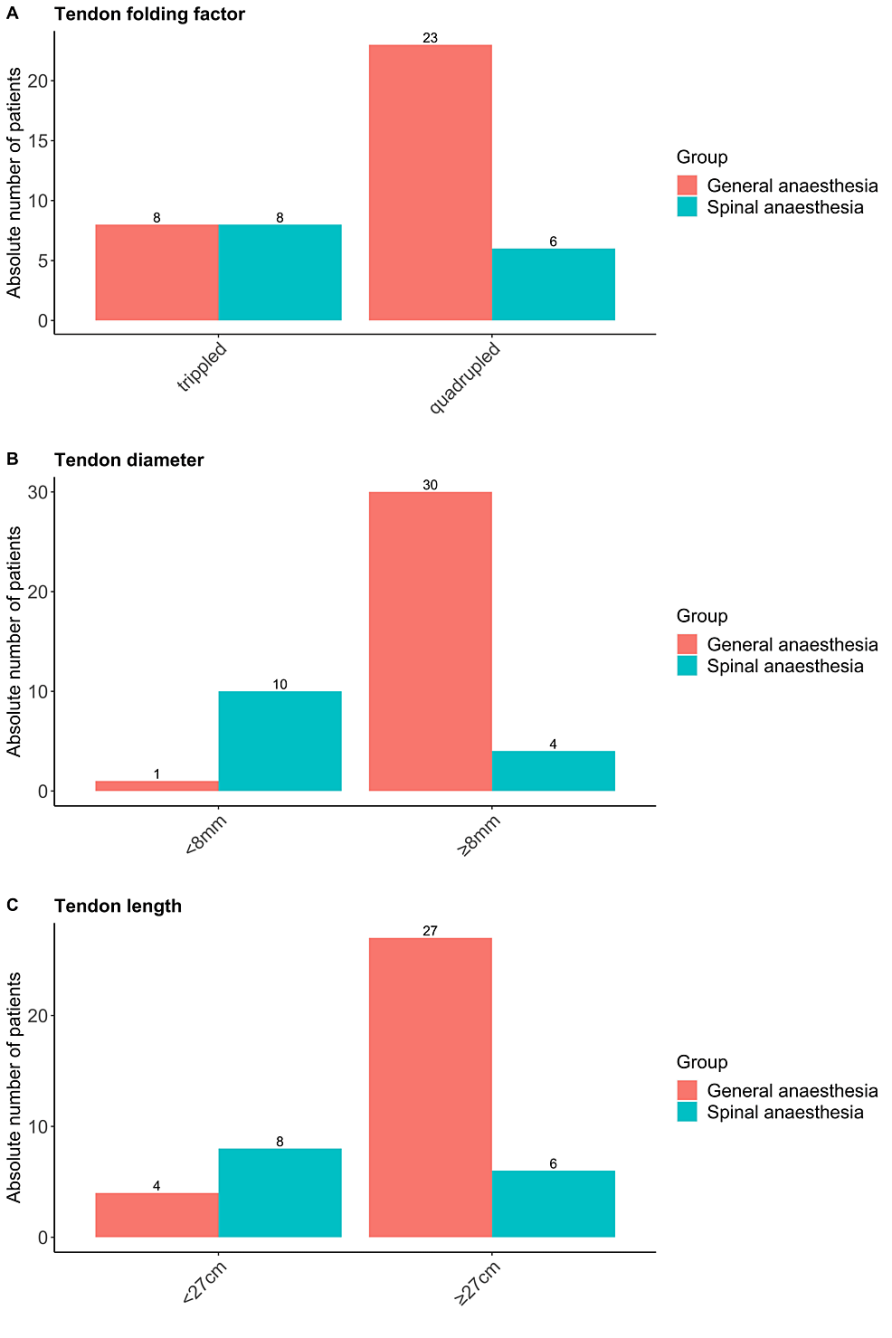
Tendon dimensions for general and spinal anesthesia cohorts Tendon folding factor (A), diameter (B), and length (C) are displayed for the general anesthesia (red) and spinal anesthesia (blue) cohorts respectively (cm=centimetre, mm=millimetre).

There were 18 men and 13 women in the GA group and four men and 10 women in the SA group (Fisher exact value 0.1075, ns). The mean age was 29.03 ± 12.44 years in the GA group and 25.43 ± 11.00 years in the SA group (p=0.34). The average height was 172.61 ± 6.21 cm in the GA group and 168.50 ± 6.73 cm in the SA group (p=0.26). The average weight 68.21 ± 12.11 kg in the GA group and 75.29 ± 13.68 kg in the SA group (p=0.11). There were no differences regarding operation time, hospital stay, or complication rate. The ST tendon length was 29.03 cm ± 2.6 cm in the GA group and 25.4 cm ± 2.70 in the SA group (t value 4.245, p=0.0001). Figure 1 summarises the results regarding the tendon dimensions.

ST tendon length was 27 cm or more in 27 out of 31 patients in the GA group and in six out of 14 patients in the SA group (p < 0.01). The ST tendon could be quadrupled in 23 out of 31 patients (83.9%) in the GA and six out of 14 patients (42.9%) in the SA group (χ2=4.13, p=0.04). The graft diameter obtained was 8.53 mm ± 0.62 mm in the GA group and 7.71 mm ± 0.47 mm in the SA group (t value 4.885, p<0.0001). Graft diameter was 8mm or more in 30 out of 31 cases (96.8%) in the GA group and in four out of 14 cases (28.6) in the SA group (χ2=24.29, p=0.000001).

## Discussion

To the best of our knowledge, this is the first study dealing with the aspect of the influence of anesthesia on the quality of an ACL graft. The issue seems to be of some importance for several reasons. There is still a lot of debate about graft dimensions in ACL replacement [1-4, 14-16]. Especially the optimal diameter of the graft and risk of failure in correlation to graft diameter is not clearly defined [4-7, 10, 17, 18]. Most publications deal with the critic diameters of an ACL graft of 7 to 8 mm [1, 2, 14, 19, 20]. Though there might be dispute about exact values the general consent “the bigger, the better” seems unchallenged [5-7, 9, 10]. There is also little disagreement regarding the fact, that harvesting the ST tendon alone is beneficial over-harvesting both hamstring tendons. Literature suggests a significantly greater loss in flexion power if ST and gracilis tendons are harvested [21, 22].

Regarding these facts, our aim was to obtain a long enough ST tendon in order to get a sufficient diameter. Obviously, this is best achieved if the tendon can be quadrupled. Furthermore, a quadrupled tendon is easier to handle for grafting, especially using the ACL tight rope® or analog techniques. Triple-strand techniques require additional graft suturing and the biomechanical properties of the third strand are still in debate [13, 23, 24]. The minimal length for a quadrupled ST tendon is not clearly defined in the literature [14, 25, 26]. Nevertheless, according to Lubowitz et al. [25], we defined the cut-off for a quadrupled tendon at 27 cm. Regarding the risk of failure, our cut-off was at a graft diameter of 8 mm. The definition of failure as well is poorly defined in studies dealing with graft dimensions [3]. It would seem, that a re-rupture caused by adequate trauma shouldn’t be considered as a failure, whereas no impairment in daily life but the inability to do sports certainly should be.

In summary of the existing arguments, our goal was clearly to get a tendon as long as possible. From a mechanical point of view, we suspected, that harvesting the tendon under certain muscle tonus will result in a shorter tendon. The amount of muscular tonus in spinal anesthesia depends on the substances used but is in any case greater than in general anesthesia [27]. To maximize the effect of general anesthesia on the muscular tonus we decided to use a short-time relaxation which was applied 10 minutes before harvesting the tendon.

From the anaesthesiologic point of view and keeping the 2018 POPULAR study’s results in mind, which found a profoundly increased risk for postoperative pulmonary complications after the use of neuromuscular blocking agents during general anesthesia [28], the choice of anaesthesiologic procedure should only be made in a multi-professional manner with regard to individual patient’s risk factors and surgical demands. But not only pre-existing pulmonary impairment, but also morbid obesity, chronic pain disorders, cognitive status, and susceptibility for peri-operative delirium are important factors to consider and weigh against the importance of a potentially longer tendon graft before giving the patient a recommendation towards a specific anaesthesiologic procedure. Combined approaches with spinal anesthesia as well as general anesthesia could be considered, too [29]. In extreme cases, one might favor an allograft.

Post-tetanic-count (PTC) measurement can be used to further quantify extraordinarily deep/intense neuro-muscular blockade when there is no more muscle contraction to the TOF stimulation used in the study. Data suggests significantly better surgical conditions when a PTC of ≤ 5/10 is ensured over a relaxation resulting only in a TOF 0/4 but PTC >5 [30]. To which extent an even longer tendon could be harvested if a profound relaxation with a PTC 0 had been administered is the subject of another study that is currently being carried out.

Limitations

The limitation of our study may be the relatively small number of patients in the spinal anesthesia group. In addition, there was no strict randomization of patients as every patient was advised to choose the kind of anesthesia freely. Furthermore, some population data or outcome-influencing factors typically found in the literature on ACL repair have been left out of this study. These include but are not limited to; athletics of population, indication for surgery, selection of patients, and time to surgery. However, as our outcome was strictly defined around the anatomical features of the semitendinosus tendon, which are not influenced by any of the factors mentioned above, we don’t think this compromises the validity of the results presented in this study. We are not trying to prove the hypothesis that a longer tendon and consequently bigger diameter have more favorable outcomes. As our literature research has concluded, this seems to be the current consensus amongst experienced ACL surgeons. On this basis, this study evaluates what method yields the longer tendon and can therefore disregard factors not directly linked to the graft measurements. For the factors we considered relevant in regard to the graft measurements, we found no significant differences between the two groups. 

## Conclusions

Applying general anesthesia with short-time relaxation while harvesting the semitendinosus tendon for ACL replacement appears to allow harvesting of a longer tendon graft leading to a bigger ACL graft diameter due to the possibility of quadrupling the tendon graft. The vast majority of experienced ACL surgeons are convinced that a greater diameter of the ACL graft is an important factor in preventing failure. Based on our findings, general anesthesia with short time relaxation appears favorable. Spinal anesthesia could be reserved for cases, where general anesthesia would be of greater risk due to the general condition of the patient. 
